# Supplementary material for: Habit formation viewed as structural change in the behavioral network
Source: Commun Biol. 2023 Apr 4;6:303. doi: 10.1038/s42003-023-04500-2 (PMC10073220; doi:10.1038/s42003-023-04500-2)
Supplement: Supplementary file 2 — Supplementary information [file 42003_2023_4500_MOESM2_ESM.pdf]

Supplementary Information

Supplementary Figures

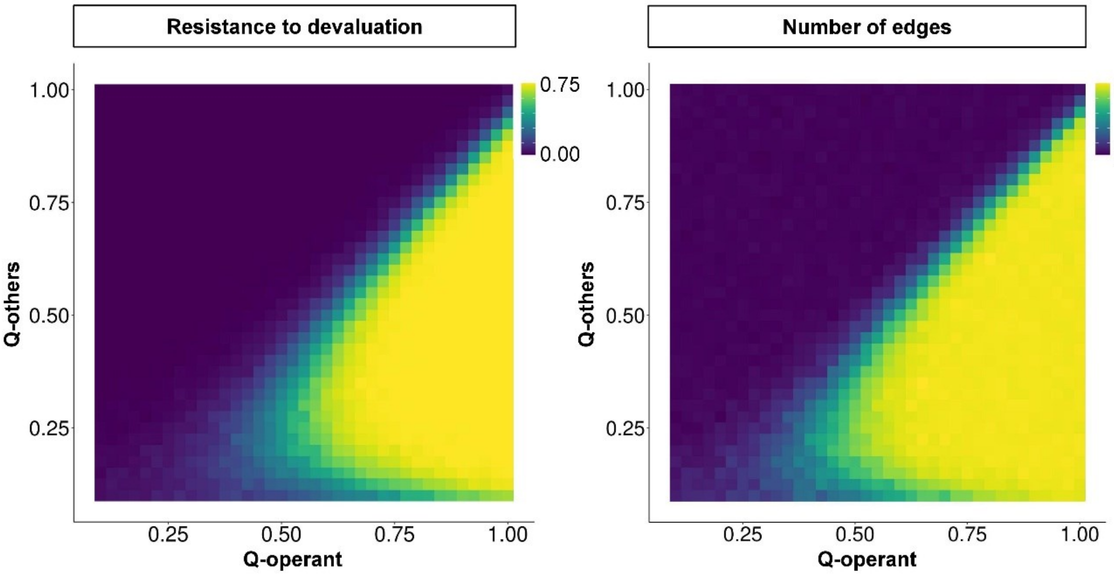

Supplementary Figure 1. Effects of systematic manipulation of $Q_{operant}$ and Q_{others} on habit formation.

The dependencies of the resistance to devaluation (left) and number of edges that the operant response acquired (right) on the $Q_{operant}$ and Q_{others}. As the $Q_{operant}$ increased, resistance to devaluation and number of edges increased, suggesting we confirmed the same result in the Simulation 1.

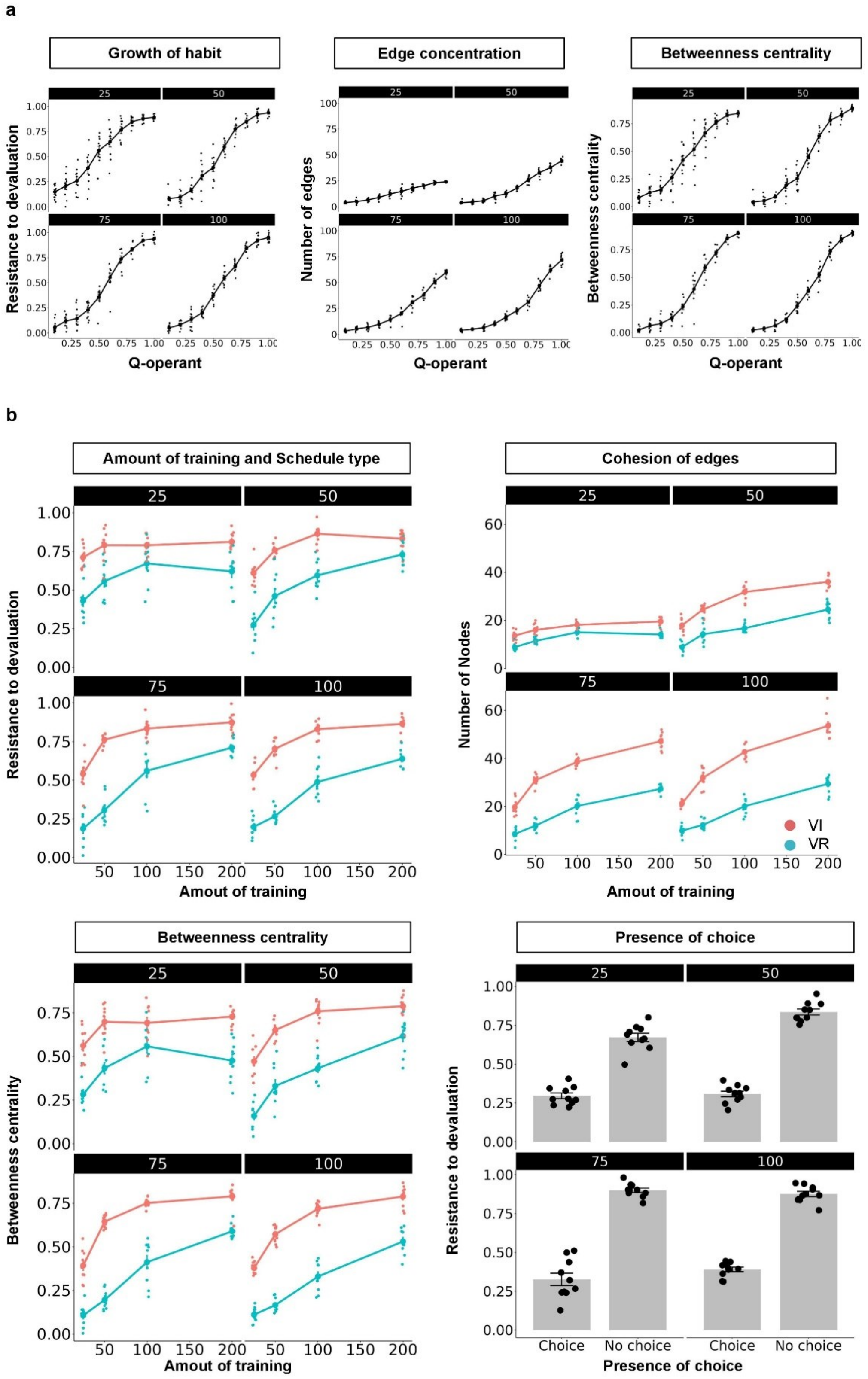
a
Growth of habit
Edge concentration
Betweenness centrality
25
50
75
100
Resistance to devaluation
Number of edges
Betweenness centrality
Q-operant
b
Amount of training and Schedule type
Cohesion of edges
Resistance to devaluation
Number of Nodes
VI
VR
Amout of training
Betweenness centrality
Presence of choice
Choice
No choice

Supplementary Figure 2. Simulation results replicating Figure 2 (simulation 1), with the different numbers of nodes (25–100).

We manipulate the number of nodes, 25, 50, 75, and 100, to confirm the results of our simulation are replicated in different numbers of nodes and all results are replicated.

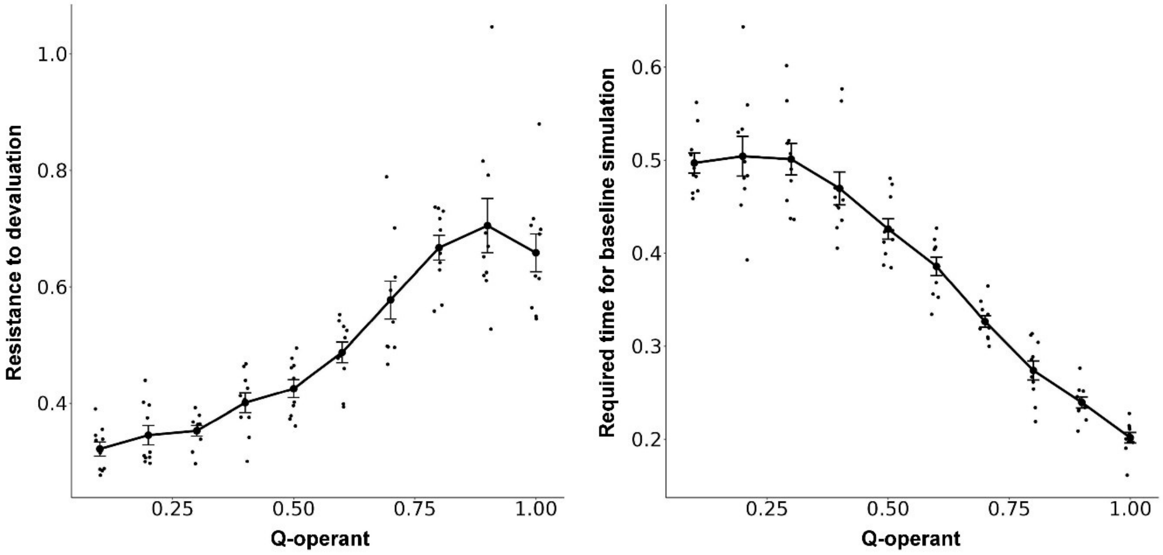

Supplementary Figure 3. Reproducibility of the results of Simulation 1 with a different response sequence generation algorithm.

In the Simulation 1, response sequences were generated by a shortest path search, Dijkstra's algorithm. We employed another algorithm that is more weakly constrained and not the shortest path searching algorithm. In the new algorithm, an agent chooses a response randomly if a response chosen as a goal is not connected to the current engaging response. If the goal response is connected to the current engaging response, the agent chooses the response. In other words, the agent searches the goal response locally in the new algorithm. Resistance to devaluation, Edge concentration and betweenness centrality, all of features are replicated with the new algorithm, suggesting habit formation does not depend on the shortest path search as long as the response sequences are generated goal-directed.

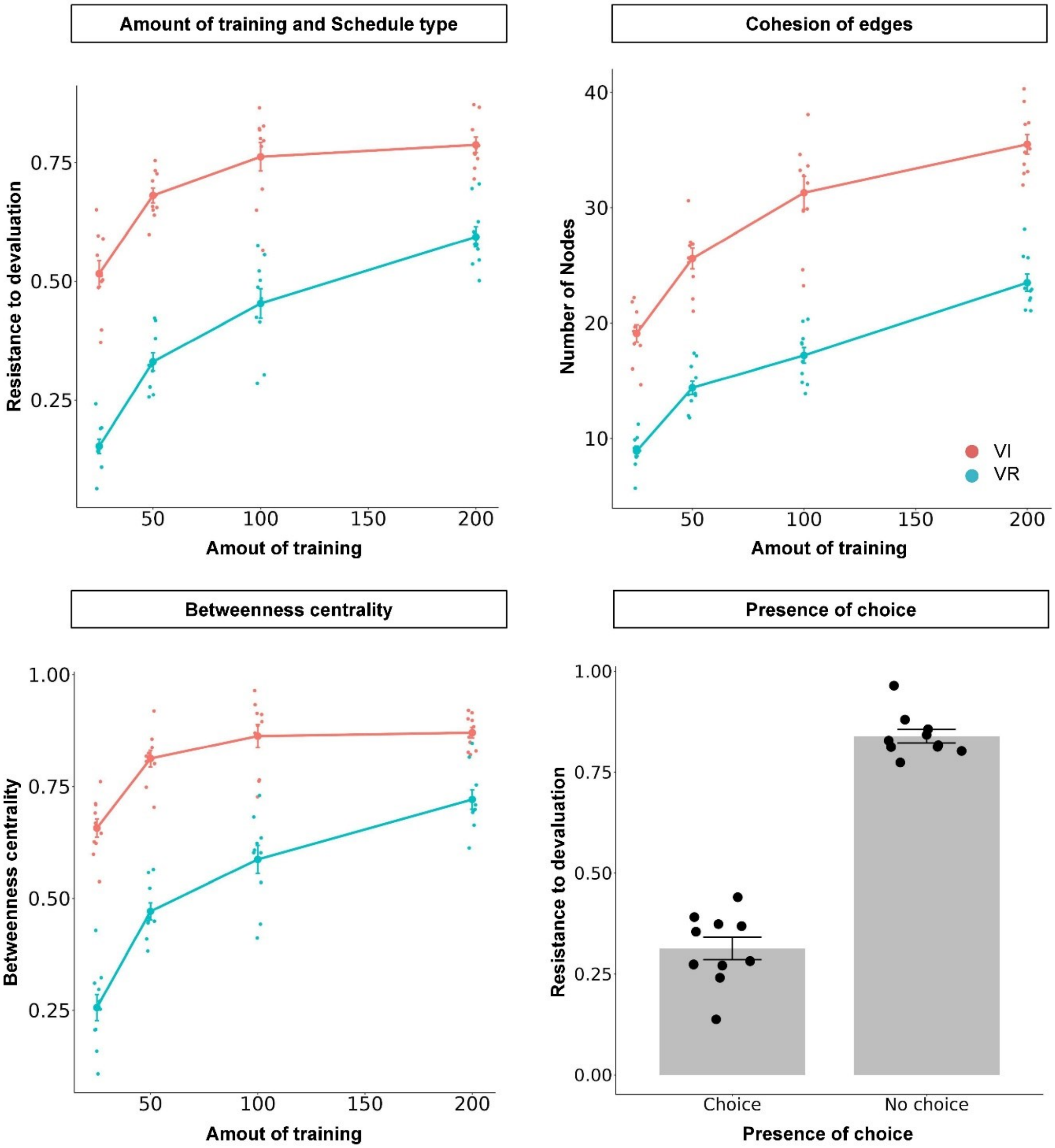

Supplementary Figure 4. Simulation results replicating Figure 3 (simulation 2), with a different schedule for other responses from the original simulation. We employed the VI 360 s schedule instead of FR 1 for other responses. We set their reward values as 1 / 50.

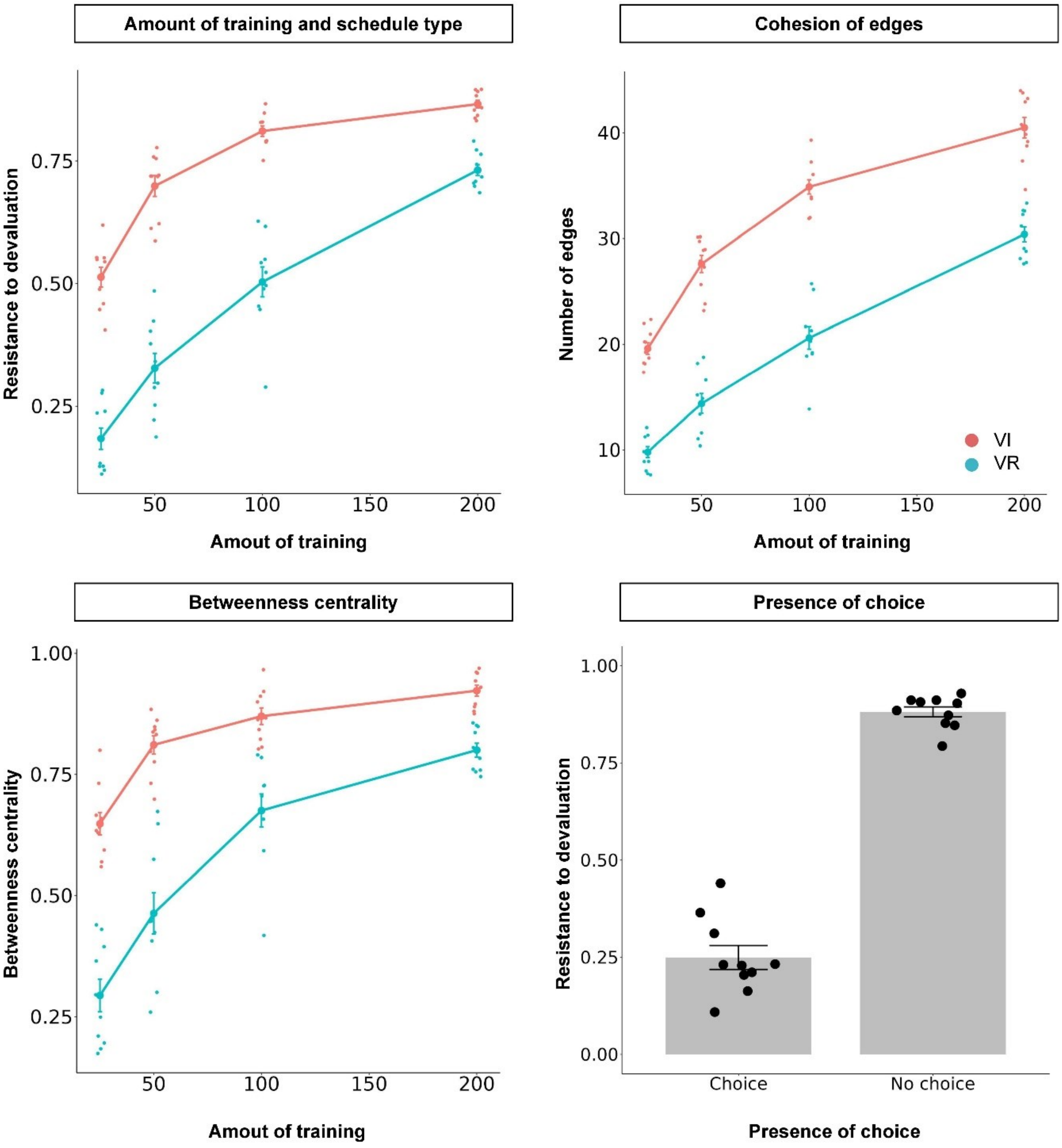

Supplementary Figure 5. Simulation results replicating Figure 3 (simulation 3), with the different algorithm SARSA from the original algorithm Q-learning.

Supplementary Methods

Algorithm 1 Learn Q-matrix in the given environment

INPUT: *N*, *schedule*, *amout_training*
OUTPUT: *Q*

Q = generate an $N \times N$ matrix with all elements set to zero.
a_{t-1} = choose a response randomly from [0, 1, 2, …, N]
response_durations = sample N samples from exponential distribution with λ = 1 / 2.5.
$r_{operant}$ = 0
while $r_{operant}$ < *amout_training*:
    a_t = choose a response with softmax function and Q-values
    τ = engage the chosen response for *response_durations*[a_t] seconds.
    r_t = schedule receive a_t and τ and return a reward
    update $Q(a_t, a_{t+1})$ according to Eq. 1
    if a_t == 0 and r_t == 1:
        $r_{operant}$ += 1
    a_{t-1} = a_t
return Q

Supplementary Algorithm 1. How agents learn Q-matrix in a given environment.

Algorithm 2 Variable ratio schedule

```
INPUT: p, rewards, amout_training
OUTPUT: schedule
q = Divide the range 0 - 1 into N equal parts
reward_count ← 0
required_response = q[reward_count] th quantile of geometry distribution with parameter p.
def schedule(a_t, _τ):
    if a_t == 0:
        required_response -= 1
    else:
        return rewards[a_t]
    if required_response <= 0:
        reward_count += 1
        required_response = q[reward_count] th quantile of geometry distribution with parameter p.
        return rewards[a_t]
return schedule
```

Supplementary Algorithm 2. The implementation of the variable ratio schedule.

Algorithm 3 Variable interval schedule

```
INPUT: λ, rewards, amout_training
OUTPUT: schedule
q = Divide the range 0 - 1 into N equal parts
reward_count = 0
required_time = q[reward_count] th quantile of exponential distribution with parameter λ.
def schedule(a_t, τ):
    required_time -= τ
    if a_t == 0 and required_time <= 0:
        reward_count += 1
        required_time = q[reward_count] th quantile of exponential distribution with parameter λ.
        return rewards[a_t]
    elif a_t != 0:
        return rewards[a_t]
return schedule
```

Supplementary Algorithm 3. The implementation of the variable interval schedule.

Algorithm 4 Concurrent VI VI schedule

```
INPUT: λ, rewards, amout_training
OUTPUT: schedule
q_1 = Divide the range 0 - 1 into N equal parts
q_2 = Divide the range 0 - 1 into N equal parts
reward_count_1 = 0
reward_count_2 = 0
required_time_1 = q_1[reward_count_1] th quantile of exponential distribution with parameter λ.
required_time_2 = q_2[reward_count_2] th quantile of exponential distribution with parameter λ.
def schedule(a_t, τ):
    required_time_1 -= τ
    required_time_2 -= τ
    if a_t == 0 and required_time_1 <= 0:
        reward_count_1 += 1
        required_time_1 = q_1[reward_count_1] th quantile of exponential distribution with parameter λ.
        return rewards[a_t]
    if a_t == 1 and required_time_2 <= 0:
        reward_count_2 += 1
        required_time_2 = q_2[reward_count_2] th quantile of exponential distribution with parameter λ.
        return rewards[a_t]
    elif a_t != 0:
        return rewards[a_t]
return schedule
```

Supplementary Algorithm 4. The implementation of the concurrent VI VI schedule.

Algorithm 5 Concurrent VI VT schedule

INPUT: *λ, rewards, amout_training*
OUTPUT: *schedule*

```
q_1 = Divide the range 0 - 1 into N equal parts
q_2 = Divide the range 0 - 1 into N equal parts
reward_count_1 = 0
reward_count_2 = 0
required_time_1 = q_1[reward_count_1] th quantile of exponential distribution with parameter λ.
required_time_2 = q_2[reward_count_2] th quantile of exponential distribution with parameter λ.
def schedule(a_t, τ):
        required_time_1 -= τ
        required_time_2 -= τ
        if a_t == 0 and required_time_1 <= 0:
                reward_count_1 += 1
                required_time_1 = q_1[reward_count_1] th quantile of exponential distribution with parameter λ.
                reward = rewards[a_t]
        elif a_t != 0:
                reward = rewards[a_t]
        if required_time_2 <= 0:
                reward_count_2 += 1
                required_time_2= q_2[reward_count_2] th quantile of exponential distribution with parameter λ.
                reward += rewards[1]
return schedule
```

Supplementary Algorithm 5. The implementation of the concurrent VI VT schedule.

Algorithm 6 Tandem VR VI schedule

```
INPUT: p, λ, rewards, amout_training
OUTPUT: schedule
q = Divide the range 0 - 1 into N equal parts
reward_count = 0
required_response = q[reward_count] th quantile of geometry distribution with parameter p.
required_time = q[reward_count] th quantile of geometry distribution with parameter λ.
def schedule(a_t, τ):
        if a_t == 0:
                required_response -= 1
        else:
                return rewards[a_t]
        if required_response <= 0:
                required_time -= τ
        if required_time <= 0. and a_t == 0:
                reward_count += 1
                required_response = q[reward_count] th quantile of geometry distribution with parameter p.
                required_time = q[reward_count] th quantile of geometry distribution with parameter λ.
                return rewards[a_t]
return schedule
```

Supplementary Algorithm 6. The implementation of the tandem VR VI schedule.

Algorithm 7 Tandem VI VR schedule

```
INPUT: p, λ, rewards, amout_training
OUTPUT: schedule
q = Divide the range 0 - 1 into N equal parts
reward_count = 0
required_response = q[reward_count] th quantile of geometry distribution with parameter p.
required_time = q[reward_count] th quantile of geometry distribution with parameter λ.
def schedule(a_t, τ):
        required_time -= τ
        if a_t == 0 and required_time <= 0:
                required_response -= 1
        elif a_t != 0:
                return rewards[a_t]
        if required_response <= 0:
                reward_count += 1
                required_response = q[reward_count] th quantile of geometry distribution with parameter p.
                required_time = q[reward_count] th quantile of exponential distribution with parameter λ.
                return rewards[a_t]
return schedule
```

Supplementary Algorithm 7. The implementation of the tandem VI VR schedule.

Algorithm 8 Procedure of baseline and devaluation phases

INPUT: *Network, N, rewards, loop*
OUTPUT: *propotion_operant*

```
s = choose an initial response from 0 - N randomly
operant = 0
total = 0
for _ in 1:loop:
        t = choose a response according to Eq. 4
        shortest_path = find a shortest path from s to t on the Network
        total += number of response contained in shortest_path
        if 0 in shortest_path:
                operant += 1
        s = t
return operant / total
```

Supplementary Algorithm 8. How agents behave in the baseline and devaluation phases.
